# Multi-Chaotic Analysis of Inter-Beat (R-R) Intervals in Cardiac Signals for Discrimination between Normal and Pathological Classes

**DOI:** 10.3390/e23010112

**Published:** 2021-01-15

**Authors:** Oleg Gorshkov, Hernando Ombao

**Affiliations:** Statistics Program, King Abdullah University of Science and Technology, Thuwal 23955, Saudi Arabia; hernando.ombao@kaust.edu.sa

**Keywords:** Lyapunov exponent, chaos, R-R interval time series, logistic regression model

## Abstract

Cardiac signals have complex structures representing a combination of simpler structures. In this paper, we develop a new data analytic tool that can extract the complex structures of cardiac signals using the framework of multi-chaotic analysis, which is based on the *p*-norm for calculating the largest Lyapunov exponent (*LLE*). Appling the *p*-norm is useful for deriving the spectrum of the generalized largest Lyapunov exponents (*GLLE*), which is characterized by the width of the spectrum (which we denote by *W*). This quantity measures the degree of multi-chaos of the process and can potentially be used to discriminate between different classes of cardiac signals. We propose the joint use of the *GLLE* and spectrum width to investigate the multi-chaotic behavior of inter-beat (R-R) intervals of cardiac signals recorded from 54 healthy subjects (hs), 44 subjects diagnosed with congestive heart failure (chf), and 25 subjects diagnosed with atrial fibrillation (af). With the proposed approach, we build a regression model for the diagnosis of pathology. Multi-chaotic analysis showed a good performance, allowing the underlying dynamics of the system that generates the heart beat to be examined and expert systems to be built for the diagnosis of cardiac pathologies.

## 1. Introduction

In modern society, heart disease is one of the major causes of mortality [1].Most clinical research in cardiology is based on the analysis of electrocardiograms (ECGs). One important characteristic of an ECG is the duration of one cardiac cycle, namely, the R-R interval. Indeed, R-R interval time series may contain information that indicates the presence of certain cardiovascular diseases. There has been considerable attention devoted to investigating the various aspects of the cardiac physiology using methods for nonlinear analysis, such as fractal analysis [2,3,4], chaos theory [5,6,7,8,9], and others [10,11]. One of the goals of such studies is to determine the most effective parameters for building expert systems for the diagnosis (and differentiation) of cardiac diseases. This is a particularly important issue, especially when creating modern portable devices for monitoring cardiac activity. Therefore, it is necessary to develop mathematical tools for non-linear analysis that can discriminate between healthy physiological and pathological R-R interval time series.

Many biomedical signals, such as cardiac signals, have complex structures representing a combination of simpler structures. These interactions reflect the influence of numerous vital processes. For example, recent studies have shown that many biomedical signals have a multifractal structure [12,13,14,15]. Such signals represent a complex fractal structure, which cannot be sufficiently characterized by a single summary value (e.g., the Hurst exponent). Multifractal signals are a combination of various simpler monofractal structures, each characterized by a single Hurst exponent. Therefore, to characterize a multifractal signal, the spectrum of generalized Hurst exponents is used, where each generalized Hurst exponent characterizes a certain monofractal structure.

**Figure 1:**
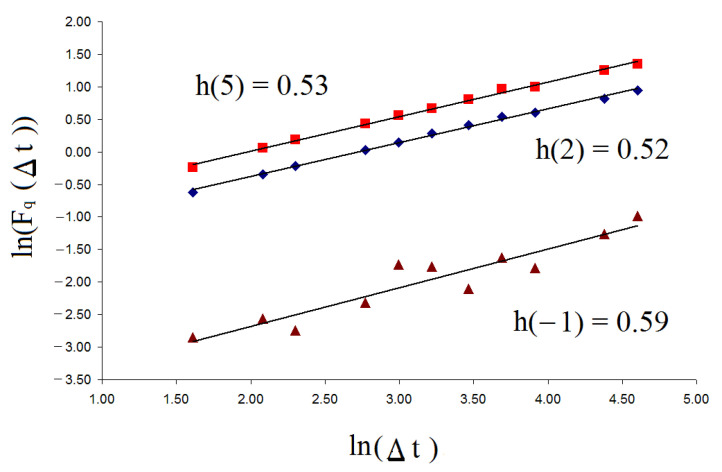
This plot shows the relationship between $\ln(F_{q}(\Delta t))$ and ln(Δt) for random time series data for *q* = −1, 2, and 5.

To obtain this spectrum from the signal, Kennel et al. [16] suggested applying the *q*-th order fluctuation function, where *q* ≠ 0. This procedure is called a multifractal detrended fluctuation analysis (MFDFA). Therefore, the MFDFA procedure for a stochastic time series {*X*(*t*)} consists of calculating the *q*-th order fluctuation function [12,16]:

(1) $$F_{q}(\Delta t)\equiv {\left(\frac{1}{\left(n-1)(\frac{N}{n}\right)}\times \sum_{j=1}^{n-1}\sum_{i=1}^{N/n}{\left[F^{2}(\Delta t)\right]}^{q/2}\right)}^{1/q},$$

where the square bias $F^{2}(\Delta t)={\left(Y((j-1)\times \Delta t+i)-P((j-1)\times \Delta t+i)\right)}^{2}$ is defined over each sliding window of interval Δt, and time series {Y(t)} is determined in the following way:

(2) $$Y_{t}=\sum_{i=1}^{t}(X_{i}-{\langle X\rangle }_{i}),$$

where ${\langle X\rangle }_{i}$ defines the cumulative moving average for $X_{1}$, …, $X_{i}$; P(x)—a line of best fit over each sliding window of length Δt; N is the number of points; and $\Delta t\;=\;\mathrm{int}(\frac{N}{n})$, where n = 2, 3, 4, …, $n_{\max}$ [12].The value $n_{\max}$ depends on the maximum size of the time series. In order to reduce the saturation effects owing to the finite size, it should be $n_{\max}<<N$ [12]. The ln–ln plot of $F_{q}(\Delta t)\sim \Delta t^{h(q)}$, as a function of Δt, yields a straight line with slope *h*(*q*), defined as the generalized Hurst exponent *h*(*q*). Figure 1 shows the ln–ln plot of $F_{q}(\Delta t)\sim \Delta t^{h(q)}$ for random time series data (white noise) for *q* = −1, 2, and 5.

The generalized Hurst exponent *h*(*q*) indicates the multifractal property of a signal. For a monofractal signal, *h*(*q*) is a constant, equaling the Hurst exponent *h*(*q*) = *H*. Conversely, for a multifractal signal, *h*(*q*) decreases as *q* increases. The singularity spectrum is assessed using the succeeding relation:

(3) α=h(q)+qh’(q) and f(α)=q[α−h(q)]+1,

where α defines the strength of a singularity spectrum and f(α) is the fractal dimension of a points set with a particular α value. For a monofractal signal, the series f(α) is converted into a single point. The measure of degree of multifractality is estimated by the width of its spectrum, which evaluates the range of α where f(α)>0:

(4) $$W=\alpha_{\max}-\alpha_{\min},$$

where $f(\alpha_{\max})=f(\alpha_{\min})=0$. Larger values for the width indicates a higher level of multifractality of the spectrum [12,16,17].

This approach is a powerful tool for analyzing biomedical signals. In [13], MFDFA was applied to a human gait time series to compare and contrast the pathology and non-pathology group. The results of the research suggested that the degree of multifractality is higher for non-pathology groups. Using the multifractal spectrum of electroencephalogram (EEG) signals, the authors of [14] developed a technique for the automated detection of epilepsy, and in [15], the analysis reveals interesting results on neural activation of the alpha (8–12 Hertz) and theta (4–8 Hertz) brain rhythms while listening to simple acoustical stimuli. This research demonstrates the ability to qualify emotions using MFDFA.

The work [12] shows the high efficiency of applying the multifractal approach for analyzing R-R interval time series. Therefore, using the *q*-th order fluctuation function, we built a two-factor logistic regression model for diagnosis of the pathology of cardiac signals with an area under the receiver operating characteristic curve (ROC curve) of 0.96 (95% CI 0.92–0.99).These results serve as inspiration for creating a four-factor logistic regression model that is able to differentiate between congestive heart failure (chf) signals from other pathologies, namely, the atrial fibrillation (af) and sudden death (sd) groups. Here, the area under the ROC curve is 0.91 (95% CI 0.84–0.97).

However, unlike fractal analysis, which permits the structure of signals to be evaluated, chaos analysis explores the base dynamics of the system, which forms the observed signal. Therefore, fractal analysis does not allow a more detailed study of the dynamic properties of the system [18]. Therefore, it is necessary to develop mathematical tools for chaotic analysis which has the power to differentiate between healthy and pathological signals (and moreover, finer differentiation across different subclasses of the pathology).

Chaotic analysis usually begins with reconstructing the phase space of a dynamical system. For reconstructing the phase space of a dynamical system, two parameters, specifically, the embedding dimension *m* and time delay *J*, are used [19,20].The time delay *J* is usually estimated either by examining the autocorrelation function (acf) [20] or mutual information (MI) [21]. The estimate of the time delay *J* is the smallest possible value that produces reconstructed attractors whose coordinates are as independent as possible (as measured by correlation or MI). Using the acf, the time delay *J* is generally chosen in accordance with the lag, where the absolute value of the acf first attains a zero (or close to it) and then flattens out. This method is quite simple and does not require massive calculations. Moreover, for Gaussian signals, a zero autocorrelation for some lag *J* or beyond implies the independence of observations at time points with absolute lag of at least *J*. However, for non-Gaussian signals, a zero correlation does not necessarily imply independence. For this reason, the delay *J* derived from the autocorrelation may produce misleading results.

Fraser and Swinney [21] have demonstrated that the MI function is a more accurate measure of independence compared to the acf. In the method proposed by Fraser and Swinney, the optimal time delay *J* is selected according to the first minimum MI function. Liebert and Schuster [22] demonstrated that the minima of the MI function match the minima of the correlation integral function, which requires less computation than the MI function [22]. Therefore, the application of the correlation integral function is more convenient for practical use.

The following approaches can be used to estimate the embedding dimension: The false nearest neighbor (FNN) method [23], Cao’s method [24], or the correlation dimension method [25,26]. The idea of the FNN method was developed using the factthat when the embedding dimension is small, points that are distant in the original phase space are brought together in the reconstruction space [24]. If *m* is defined as an embedding dimension by the embedding theorems [27,28], then the *true neighbors* are any two points which are close in the *m*-dimensional reconstructed space and that remain close in the (*m* + 1)-dimensional reconstructed space. On the contrary, there are *false neighbors*. However, the FNN method is not accurate enough to determine the parameters $R_{tol}$ and $A_{tol}$.Therefore, this approach may lead to incorrect estimation of the embedding dimension. Cao’s method [24] overcomes the shortcomings of the nearest neighbor method, which makes it more attractive for practical applications such as cardiac signals.

One of the conditions for the chaotic state of a dynamic system is sensitivity to the values of the initial conditions [19,20]. The largest Lyapunov exponent (*LLE*) is often considered as one quantitative measure of this sensitivity. The largest Lyapunov exponent characterizes the degree of exponential divergence of close trajectories [20]. The presence of a positive Lyapunov exponent in the system indicates that any two close trajectories quickly diverge over time, that is, there is sensitivity to the values of the initial conditions. Therefore, determination of the Lyapunov exponent recognizes the existence of chaotic behavior [19,20].

It is understood that many biomedical signals have a complex chaotic structure, which is the result of the interaction of various chaotic systems involved in the regulation of vital processes in the body. This complex structure is a combination of simpler chaotic structures. To calculate the characteristic values of these chaotic structures, the non-Euclidean norms can be used. The family of Minkowski norms [29,30] are parameterized by their exponents *p* = 1,2,…:

(5) $$D_{x,y}={\left(\sum_{i}{\left|\left(x_{i}-y_{i}\right)\right|}^{p}\right)}^{1/p}.$$

Generalizations of Minkowski norms are presented in [29] for the event where *p* is a positive real number. For *p* ≥ 1, those extensions are actually norms, but for 0 < *p* < 1, the triangle inequality does not remain and they cannot be regarded as norms. Therefore, *p*-norms with *p* < 1 were named fractional norms. Below, we denote the Minkowski norm and a fractional norm as *p*-norm ${\left\|\right\|}_{p}$.

In this paper, to characterize the chaotic structure, we propose only considering the largest Lyapunov exponent. Therefore, we will not consider the entire spectrum of values of the Lyapunov exponent for a given chaotic structure, calculated for the corresponding *p*-norm ${\left\|\right\|}_{p}$, which will greatly simplify the calculations. Each *p* value of *p*-norm ${\left\|\right\|}_{p}$ will correspond to its largest Lyapunov exponent *LLE(p).* Therefore, a complex chaotic structure will be characterized by the spectrum of *LLE(p)* for *p*∈(0;+∞). If the entire spectrum of *LLE(p)* values is characterized by a single value, then this structure will have mono-chaotic behavior. If some variability of the *LLE(p)* values is observed, then the considered structure is said to possess multi-chaotic behavior.

We believe that the R-R interval time series is a combination of various chaotic structures that results in the interaction with the regulation of cardiac activity. To evaluate the chaotic structure, we propose estimating the largest Lyapunov exponent using the *p*-norm ${\left\|\right\|}_{p}$, based on the well-known method proposed by Rosenstein et al. in [20].This approach can allow us to identify the multi-chaotic behavior of R-R intervals. Consequently, we anticipate improvement in the differentiation of the chaotic properties of R-R intervals for healthy and non-healthy subjects compared to the standard approach, proposed by Rosenstein et al. in [20]. To demonstrate the effectiveness of the new approach, we will construct logistic models for differentiating R-R intervals for healthy and non-healthy subjects. In summary, the goal of this study is to design a new approach for identifying the multi-chaotic behavior of time series and demonstrate its effectiveness for evaluating the multi-chaotic properties of R-R intervals.

## 2. Materials and Methods

### 2.1. Clinical Datasets

In 2008, the editors of Chaos proposed the following research question: “Is the Normal Heart Rate Chaotic?” [31]. For this study, PhysioNet [32] provided the records of R-R intervals in the case of healthy subjects (hs) and patients with congestive heart failure (chf) and atrial fibrillation (af). Since our research deals with this issue, we used the proposed groups for exploring the multi-chaotic properties of R-R intervals. Therefore, we explored the cardiac time series, producing 5000 points (≈1 h) for the 24h R-R interval time series of 54 hs (Normal Sinus Rhythm R-R Interval Database), 44 chf (Congestive Heart Failure Database), and 25 af (MIT-BIN Atrial Fibrillation Database).

### 2.2. Statistical Methods

The Rosenstein method is a very popular approach for evaluating the largest Lyapunov exponent of biomedical signals [20]. The algorithm outline is as follows. Let us consider a stochastic time series {X(t)}. The reconstructed trajectory, ${\vec{X}}_{r}$, is presented as a matrix where each row is a phase-space vector:

(6) $${\vec{X}}_{r}={\left({\vec{X}}_{r1}{\vec{X}}_{r2}\ldots {\vec{X}}_{rM}\right)}^{T},$$

where ${\vec{X}}_{ri}$ is the state of the system at discrete time *i*. For an *N*-point time series of {$x_{1},x_{2},\ldots ,x_{N}$}, each ${\vec{X}}_{ri}$ is defined by

(7) $${\vec{X}}_{ri}=\left(x_{i},x_{i+J}\ldots x_{i+(m-1)J}\right),$$

where *J* is the time delay (lag), and *m* is the embedding dimension. Thereby, ${\vec{X}}_{r}$ is an M×m matrix, and the constants *m*, *M*, *J*, and *N* are related as M=N−(m−1)×J [20].

Rosenstein et al. [20] assumed that the jth pair of nearest neighbors approximately diverge at a rate presented by the largest Lyapunov exponent LLE:

(8) $$d_{j}(i)=C_{j}e^{LLE(i\times \Delta t)},$$

where $d_{j}(i)$ is the distance between the jth pair of nearest neighbors after *i* discrete-time steps, Δt is the sampling period of the time series, and $C_{j}$ is the initial separation. The largest Lyapunov exponent LLE is evaluated by a linear approximation of the *average* line determined by

(9) $$y(i)=\frac{1}{\Delta t}\langle \ln d_{j}(i)\rangle ,$$

where 〈…〉 designates the average overall values of *j*. In Rosenstein et al. [20], $d_{j}(i)$ is defined as follows:

(10) $$d_{j}(i)=\underset{{\vec{X}}_{j_{\min}}}{\min}{\left\|{\vec{X}}_{j+i}-{\vec{X}}_{j_{\min}}\right\|}_{}=\underset{{\vec{X}}_{j_{\min}}}{\min}((x_{j+i}-x_{j_{\min}}){}^{2}+\;(x_{j+i+J}-x_{j_{\min}+J}){}^{2}\;+\ldots \;+\;(x_{j+i+(m-1)J}-x_{j_{\min}+(m-1)J}){}^{2}){}^{1/2},$$

where ‖‖ denotes the Euclidean norm.

Many biological time series have a complex structure in which the interweaving of several chaotic structures can be observed. These time series cannot be characterized by a single *LLE* value; and characterization requires a spectrum of *LLE* values measuring each chaotic structure. Obviously, this spectrum will be characterized by a certain spectrum width *W*, which will reflect the degree of multi-chaos of the studied process. Therefore, the width *W* of the spectrum will be zero for a mono-chaotic series. Larger values of the width *W* indicate a higher degree of multi-chaos in the time series. To estimate this spectrum, we propose a new approach, which is based on a generalization of the Rosenstein method, used for estimating the largest Lyapunov exponent. We call this approach a multi-chaotic analysis. Therefore, if the time series is characterized by a certain spectrum of *LLE* (accordingly, there is some variation of *LLE*), then it has a multi-chaotic behavior. Conversely, if the time series is characterized by a single *LLE* value, then it has mono-chaotic behavior.

The application of *p*-norms ${\left\|\right\|}_{p}$ allows the spectrum of *LLE* values that define the rate of divergence of the jth pair of nearest neighbors to be determined for each *p*-norm. The distance between the two vectors ${\vec{X}}_{j+i}$ and ${\vec{X}}_{j}$ in an m-dimensional space for *p*-norm ${\left\|\right\|}_{p}$ is calculated as(${\left|x_{j+i}-x_{j}\right|}^{p}$+${\left|x_{j+i+J}-x_{j+J}\right|}^{p}$+…+${\left|x_{j+i+(m-1)J}-x_{j+(m-1)J}\right|}^{p}$)${}^{1/p}$, where *p* > 0. Then, the distance between the jth pair of nearest neighbors in the m-dimensional space for *p*-norm after *i* discrete-time steps is defined as follows:

(11) $$d_{j}^{p}(i)=\underset{{\vec{X}}_{j_{\min}}}{\min}{\left\|{\vec{X}}_{j+i}-{\vec{X}}_{j_{\min}}\right\|}_{p}=\;\underset{{\vec{X}}_{j_{\min}}}{\min}({\left|x_{j+i}-x_{j_{\min}}\right|}^{p}+{\left|x_{j+i+J}-x_{j_{\min}+J}\right|}^{p}+\ldots \;+{\left|x_{j+i+(m-1)J}-x_{j_{\min}+(m-1)J}\right|}^{p}){}^{1/p}.$$

We denote these largest Lyapunov exponents as the generalized largest Lyapunov exponents (*GLLE*). Therefore, the jth pair of nearest neighbors for *p*-norm ${\left\|\right\|}_{p}$ will approximately diverge at a rate presented by the GLLE:

(12) $$d_{j}^{p}(i)=C_{j}e^{GLLE(i\times \Delta t)}.$$

The generalized largest Lyapunov exponent is evaluated by a linear approximation of the *average* line determined by

(13) $$y_{p}(i)=\frac{1}{\Delta t}\langle \ln d_{j}^{p}(i)\rangle .$$

From Equation (11), it is clear that using values *p* < 1 makes it possible to enhance the influence of small fluctuations (SF) between the coordinates of the vectors ${\vec{X}}_{j+i}$ and ${\vec{X}}_{j}$ on the estimate of the distance $d_{j}^{p}(i)$ compared to the Euclidean norm distance $d_{j}(i)$. On the contrary, using values *p* >> 1 makes it possible to enhance the influence of large fluctuations (LF) between the coordinates of the vectors ${\vec{X}}_{j+i}$ and ${\vec{X}}_{j}$ on the estimate of the distance $d_{j}^{p}(i)$ compared to the Euclidean norm distance $d_{j}(i)$.In this way, we can obtain a filter that enhances the contribution of the SF or LF component to the evaluation of the distance $d_{j}^{p}(i)$, according to an evaluation of the generalized largest Lyapunov exponent. Therefore, multi-chaotic behavior is characterized by the difference between SF and LF components of the distance $d_{j}^{p}(i)$ between the two vectors ${\vec{X}}_{j+i}$ and ${\vec{X}}_{j}$ in them-dimensional space. The difference between SF and LF components determines the spectrum width *W*.

Now consider the results of applying this approach to well-known chaotic dynamical systems. Table 1 demonstrates the chaotic dynamical systems that were used to evaluate the *GLLE(p)* of the proposed approach. The fifth column presents the theoretical values of the largest Lyapunov exponent. By applying fourth-order Runge–Kutta integration, the differential equations were solved numerically for using the x-coordinate time series to reconstruct the dynamics.

**Figure 2:**
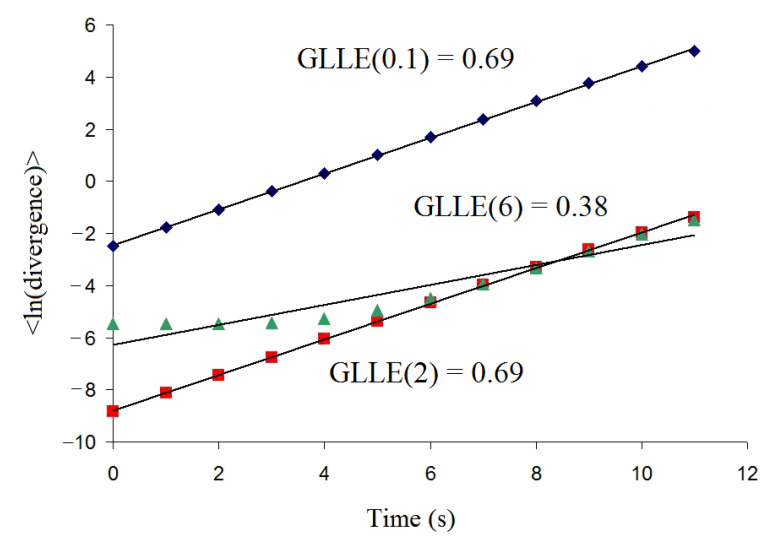
The dependencies of<ln(divergence)> versus time for the logistic map for *p*-norm = 0.1, 2, and 6.

Figure 2 demonstrates the relationship between $\langle \ln d_{j}^{p}(i)\rangle$ and i×Δt for the logistic map for *p-norm* = 0.1, 2, and 6, where “<ln(divergence)>” denotes $\langle \ln d_{j}^{p}(i)\rangle$ and “Time(s)” corresponds to i×Δt.

Table 2 shows the calculation results of *GLLE(p)* for the Logistic, Henon, Lorenz, and Rossler chaotic dynamical systems using the *p*-norm ${\left\|\right\|}_{p}$. To estimate the spectrum width *W*, we used the difference between the maximum and minimum values of *GLLE*: Δ*W = GLLE* ${}_{\max}$*− GLLE*${}_{\min}$.

Table 2 demonstrates that for *p*-norm < 1, decreasing the *p*-norm leads to a decrease of *GLLE(p)*. Apparently, this is due to the fact that increasing the SF component leads to deterioration in the chaotic properties of the considered attractors. At the same time, for *p*-norm > 1, we can see that increasing the *p*-norm leads to a decrease in *GLLE(p)* for the Logistic and Henon maps. The reason for this is the deterioration of the chaotic properties of attractors with an increasing LF component. A similar effect is observed for the Lorenz attractor; however, some oscillations of *GLLE(p)* are present, which we associate with the structure of the attractor. Therefore, the different influence of SF and LF components leads to estimation of the spectrum width Δ*W* > 0. This indicates the multi-chaotic behavior of the Logistic map, the Henon map, and the Lorenz attractor.

We can see that *GLLE(p)* of the Rossler attractor reaches a plateau for *p*-norm > 6 and the estimation of the spectrum width Δ*W* ≈ 0. This indicates the mono-chaotic behavior of the Rossler attractor. It is obvious that the application of the presented method is not advisable for mono-chaotic behavior. Therefore, it can be seen that the *GLLE(p)* of attractors can exhibit different behaviors; therefore, we recommend preliminarily estimating the interval of *p*-norm values. It should be noted that at close to zero *p*-norm values, the *GLLE(p)* values become extremely unstable. At the same time, when the *p*-norm has a large value, *GLLE (p)* becomes close to zero and loses its information content.

Since the presence of a random component is characteristic of biological signals (i.e., most biological signals are not perfectly deterministic), we consider the influence of a random component on the spectrum width *W*. Table 3 shows the results of the estimation of the spectrum width Δ*W* for chaotic dynamical systems, obtained by adding a random component to the x-coordinate time series of the aforementioned chaotic dynamical systems. The random components have a normal distribution with a zero mean and standard deviations presented in Table 3.

Table 3 shows that increasing the standard deviation of the random component will lead to a decrease of the estimation of the spectrum width Δ*W*. Therefore, when increasing a random component of the signal, the spectrum width *W* decreases. An exception is the Rossler attractor, which is characterized by mono-chaotic behavior. Obviously, in this case, the values of the spectrum width Δ*W* remain constant.

## 3. Results

We will now investigate the feasibility of applying multi-chaotic analysis to reveal any possible difference among R-R interval time series of healthy subjects (hs), subjects diagnosed with congestive heart failure (chf), and subjects diagnosed with atrial fibrillation (af). To estimate the delay time *J* along with the widely used methods of the acf and the MI function, we used the correlation integral function $C_{m}(J)$, derived from [20]. The embedded dimension *m* was evaluated by applying the method in [24]. Appendix A contains graphs demonstrating the results of applying this method for one of the R-R intervals recorded. Preliminary estimation of the interval of *p*-norm values showed us that *p*∈[0.1; 5] is the most appropriate interval for assessing the *GLLE (p)* of R-R interval time series.

**Figure 3:**
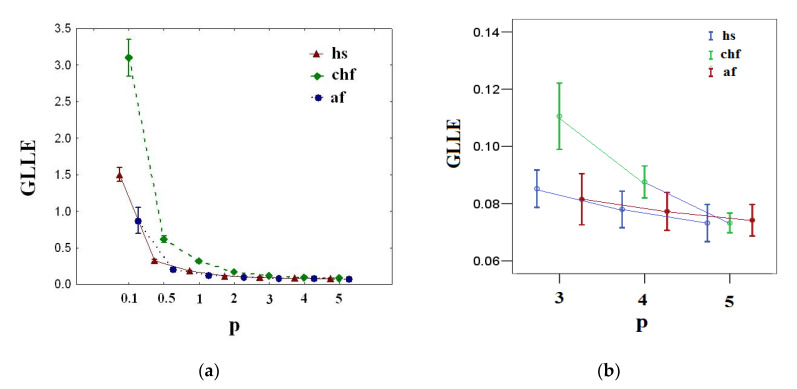
(**a**) Generalized largest Lyapunov exponents *GLLE(p)* vs. order *p* for healthy (hs), congestive heart failure (chf), and atrial fibrillation (af) groups. (**b**) The same values for *p* = 3, 4, and 5. The error bars represent the standard error of the mean.

Figure 3 shows the results of calculating *GLLE(p)* for healthy (hs), congestive heart failure (chf), and atrial fibrillation (af) groups.

From Figure 3a, it is clear that there is variation of *GLLE(p)* with *p* for healthy (hs), congestive heart failure (chf), and atrial fibrillation (af) groups, where the values of *GLLE(p)* decrease with increasing *p*. This indicates the multi-chaotic behavior of the R-R interval time series considered. Figure 3b demonstrates that there is no statistically significant difference (*p*-value > 0.05) among groups for *GLLE (5)*. Therefore, for multi-chaotic analysis of the considered signals, it is sufficient to use *p*-norm ≤5.

To characterize multi-chaotic behavior, let us consider estimation of the spectrum width Δ*W = GLLE*${}_{\max}$−*GLLE*${}_{\min}$. Since standard approaches analyze chaotic behavior using the Euclidean norm, given the importance of this issue for the chaotic analysis of R-R interval time series, we separately consider the values of the largest Lyapunov exponent *GLLE(2).* Table 4 shows the results achieved for the largest Lyapunov exponent *GLLE(2)* and estimation of the spectrum width Δ*W*. Since the empirical distribution of the estimated values differs from a normal distribution (*p*-value < 0.05, Kolmogorov–Smirnov test), we proceeded to use the median (*Me*) in order to evaluate the central values of *GLLE(2)* and Δ*W*, and applied the first quartile $Q_{1}$ and third quartile $Q_{3}$ to evaluate the variation or spread of the distribution of the considered values.

From Table 4, note that the chf group *GLLE(2)* = 0.15(0.12–0.20) is characterized by higher values (*p*-value < 0.05, ANOVA F-test) than hs *GLLE(2)* = 0.11(0.10–0.13). The af group *GLLE(2)* = 0.08(0.07–0.10) has lower values (*p*-value < 0.05, ANOVA F-test) compared to the hs group *GLLE(2)*. These results clearly demonstrate that for the Euclidean norm, the chf heart rate control system is more sensitive to the initial conditions, whereas the af heart rate control system is less sensitive to the initial conditions compared to the hs heart rate control system. Nevertheless, Figure 3b demonstrates that when increasing the *p*-norm, these differences degrade, which indicates the important role of the SF component in the differentiation of the R-R interval time series.

Table 4 shows that pathology can be characterized by different spectral widths. Using the ANOVA method, we conclude that the spectrum width of the congestive heart failure group Δ$W_{chf}$ = 2.61(1.70–4.23) is significantly greater (*p*-value < 0.05, ANOVA F-test) than each ofthe healthy group Δ$W_{hs}$ = 1.28(0.78–1.95) and atrial fibrillation group Δ$W_{af}$ = 0.42(0.26–0.79). The spectrum width Δ$W_{af}$ for the atrial fibrillation group has significantly lower values (*p*-value < 0.05, ANOVA) than the healthy group Δ$W_{hs}$.Therefore, the R-R interval time series of the chf group is characterized by the largest difference between the SF and LF components, and the af group is characterized by the smallest difference. This result leads us to make an assumption about the different level of the random component in the groups of the studied signals. Therefore, we can infer from these findings that the random component dominates in the af R-R interval time series compared to the hs R-R interval time series. On the contrary, the contribution of the random component in the congestive heart failure R-R interval time series is smaller compared to in the hs R-R interval time series intervals.

It is widely accepted that a good indicator of the level of the random component of a chaotic signal is the correlation dimension $D_{2}$. Argyris et al. [33] demonstrated that the correlation dimension increases when increasing the level of the random component of the chaotic signal. We now use this property of the correlation dimension to test our assumption about the level of the random component in healthy, congestive heart failure, and atrial fibrillation groups. Table 5 shows the results achieved for the correlation dimension $D_{2}$.

Table 5 demonstrates that the correlation dimension of the af group $D_{2}$= 0.93(0.69–1.00) is significantly greater (*p*-value < 0.05, ANOVA F-test) than each of the hs group $D_{2}$ = 0.57(0.50−0.66) and chf group $D_{2}$ = 0.55(0.17−0.85). This confirms our assumption that the variance contribution of the random component is larger in the af group R-R interval time series compared to the hs group R-R interval time series. However, no statistically significant difference (*p*-value < 0.05, ANOVA F-test) was found between the correlation dimension of the hs and chf groups.

By setting up a logistic regression model [34], we explored the capacity of multi-chaos analysis to discriminate between healthy group R-R interval time series and the pathological group R-R interval time series. Predictors of the logistic regression model were the attributes (GLLE_p) GLLE_0.1, GLLE_0.5, GLLE_1, GLLE_2, GLLE_3, GLLE_4, and GLLE_5. Considering that the input parameters of the model have a small range of changes, we multiplied them by a hundred. Therefore, the odds ratio can be estimated more efficiently. The data were separated into the training set (85 cases) and the testing set (38 cases). The output variable is denoted as Y = 0 if the subject belongs to the healthy group and, accordingly, Y = 1 if the subject belongs to the pathology group. To choose the minimum set of factor attributes, a stepwise-variable selection (SVS) method was applied [12,34]. Therefore, five attributes, introduced in Table 6, were selected. The estimated parameters of the logistic regression model (model-1) for the log odds that a signal belongs to the pathology group is given by

(14) $$\ln\left(\frac{P}{1-P}\right)=-0.45\;+\;1.02\;\times \;\mathrm{GLLE}\_0.1\;-\;5.18\;\times \;\mathrm{GLLE}\_0.5\;-\;7.09\times \;\mathrm{GLLE}\_1\;+\;105.39\;\times \;\mathrm{GLLE}\_4\;-\;95.23\;\times \;\mathrm{GLLE}\_5,$$

where *P* is the probability of belonging to the pathology group.

Table 6 indicates that an increase of one unit in the GLLE_0.1 (from baseline) indicates that the odds of being in the pathology group is 2.78 times the odds at the baseline, keeping the other factors constant. Similarly, increasing GLLE_4 by 1.0 unit leads to an increased odds of belonging to the pathology group. On the contrary, keeping all attributes constant, increasing GLLE_0.5, GLLE_1, or GLLE_5 by 1.0 unit leads to an increased odds of being in the healthy group.

**Figure 4:**
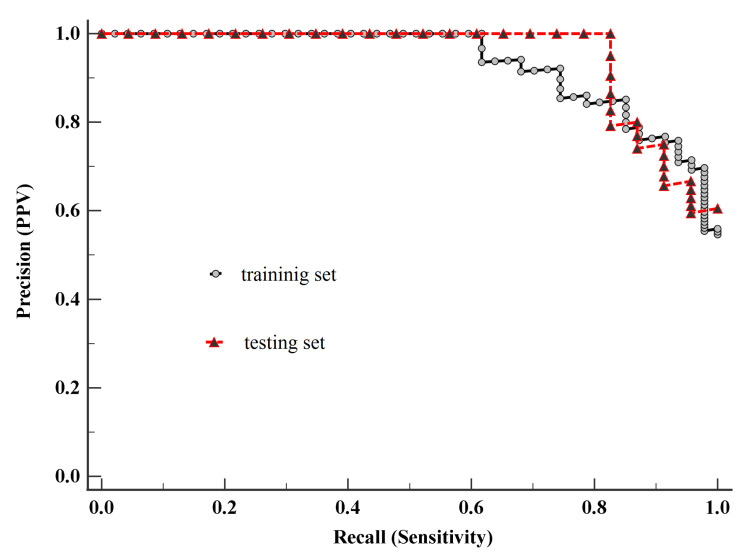
The Precision-Recall Curves of model-1, building on the training set and the testing set.

Since we had unbalanced samples, we used the Precision-Recall Curve (PRC) to evaluate the performance of the classifier [35]. Figure 4 demonstrates the PRC curve of model-1 built on the training set. The area under the PRC curve (AUC) equals 0.93 (95% CI 0.88–0.97), suggesting a good performance. Analyzing the PRC curve allowed us to determine the associated criterion, defining the threshold of model-1, which is greater than 0.46.

Taking into account the values of the associated criterion and Equation (14), classification results of model-1 for the training and testing sets were determined. The classification results of model-1 are given in Table 7.

To assess the performance of the classifier, the Matthews correlation coefficient (MCC) [35] was calculated, based on the training set, to be MCC= 0.65 and based on the testing set, to be MCC = 0.57. These results indicate the good quality of the model in terms of both the training set and testing set. Furthermore, evaluating the quality of the model, its PRC curve was constructed based on the testing set. This PRC curve is shown in Figure 4, where AUC equals 0.95 (95% CI 0.88–0.98), demonstrating a good performance. Since that there is no statistically significant difference (*p*-value > 0.05) between the AUC of the PRC curve built on the testing set and the AUC of PRC curve built on the training set, it can be assumed that this model has the potential to be used for diagnostic purposes. Nevertheless, for use in diagnostics, this model requires additional research based on a larger sample.

Given the difference in multi-chaotic behavior in the RR intervals time series of the chf and af, the next step is to identify the values that differentiate between the congestive heart failure and the atrial fibrillation groups. Unfortunately, due to the small sample size, we did not split the sample into a training and testing set. Therefore, the results of this model are preliminary and require further additional research with a larger sample size.

By constructing a logistic regression model as a classifier based on the attributes (GLLE_p) GLLE_0.1, GLLE_0.5, GLLE_1, GLLE_2, GLLE_3, GLLE_4, and GLLE_5, we defined statistically significant values and assessed their impact. To avoid uncertainty in the form of infinity, we multiplied the attributes by a hundred. The output variable is denoted by Y = 0 for the congestive heart failure group and, accordingly, Y = 1 for the atrial fibrillation group. To choose the minimum set of factor attributes, the SVS method was applied [12,34]. Consequently, three factor values were identified, which are introduced in Table 8.The estimated parameters of the logistic regression model (model-2) for the log odds that a signal belongs to the atrial fibrillation group are given by

(15) $$\ln\left(\frac{P}{1-P}\right)=3.34\;-\;4.851\;\times \;\mathrm{GLLE}\_1\;+\;75.71\;\times \;\mathrm{GLLE}\_4\;-\;70.84\;\times \;\mathrm{GLLE}\_5.$$

Table 8 indicates that an increase of one unit in the GLLE_4 (from the baseline) indicates that the odds of being in the atrial fibrillation group is 7.62 × 10${}^{32}$ times the odds at the baseline, keeping the other factors constant. On the contrary, when keeping all attributes constant, increasing GLLE_1 or GLLE_5 by 1.0 unit leads to the odds of being in the congestive heart failure group being increased.

**Figure 5:**
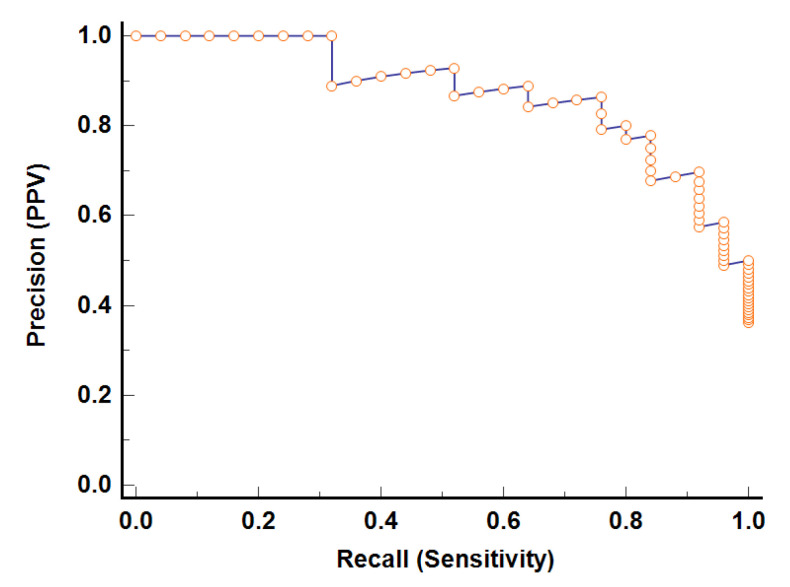
The Precision-Recall Curve of model-2.

Figure 5 shows the PRC curve of model-2. The area under the PRC curve equals 0.87 (95% CI 0.74–0.95), demonstrating good performance.

The associated criterion, defining the threshold of model-2, is greater than 0.48. In view of the values of the associated criterion and Equation (15), the classification results of model-2 were defined. The classification results of model-2 are given in Table 9.

The Matthews correlation coefficient equals 0.68. These results indicate the good performance of model-2. Therefore, the preliminary model demonstrated the good potential of the proposed approach to discriminate between the congestive heart failure and atrial fibrillation patients. However, this study should be continued with a larger sample.

## 4. Discussion

We developed a new method for analyzing and evaluating the behavior of nonlinear time series. This method is based on the assumption that some biological time series have a complex chaotic structure, which is formed of a combination of simpler chaotic structures. These types of time series cannot be adequately characterized by a single *LLE* value, but have to be characterized by a spectrum of *LLE* values. To determine this spectrum of *LLE* values, *p*-norms ${\left\|\right\|}_{p}$ were used. The application of *p*-norms ${\left\|\right\|}_{p}$ allows the contribution of the SF component or LF component to the estimate of the distance $d_{j}^{p}(i)$ between the jth pair of nearest neighbors in the m-dimensional space to be strengthened. Obviously, a larger difference between SF and LF components indicates a more complex chaotic structure of the studied signal. The difference between SF and LF components defines the spectrum width *W*.

As a result of using this approach for analyzing the Logistic map, Henon map, and Lorenz attractor, we note that these attractors produce the estimation of the spectrum width of Δ*W* > 0 and, accordingly, are characterized by multi-chaotic behavior. However, the Rossler attractor, having Δ*W* ≈ 0, is characterized by mono-chaotic behavior. Therefore, the time series can be characterized by mono-chaotic or multi-chaotic behavior.

Since the biomedical signal is a product of an interaction of a large number of different biological systems, this leads to the presence of a random component in the biomedical signal. Therefore, we conducted studies on the influence of the random component on the multi-chaotic behavior of the investigated chaotic systems. As a result, we found that as the variance contribution of the random component increases, the spectrum width *W* decreases.

This approach has been applied to discover possible differences among R-R interval time series of healthy subjects (hs), subjects diagnosed with congestive heart failure (chf), and subjects diagnosed with atrial fibrillation (af).As a result of the study, it was found that for the Euclidean norm, the heart rate control system in chf subjects is more sensitive to the initial conditions than in hs subjects. Moreover, for the Euclidean norm, the heart rate control system in af subjects is less sensitive to the initial conditions than in hs subjects. We believe that these distinctions are related to the difference in the chaos control strategy [7].

Studies have demonstrated that estimation of the spectrum width ΔW can be used as one of the parameters to evaluate the level of the random component of the chaotic biomedical signal. As a result of evaluating the spectrum width ΔW and the correlation dimension $D_{2}$, it was found that the contribution of the random component in the atrial fibrillation group R-R interval time series is larger compared to in the hs R-R interval time series and chf R-R interval time series.

By applying multi-chaos theory, we built a five-factor logistic model that is able to distinguish the hs signal from the pathological groups, i.e., the chf and af groups. This model has been demonstrated to have a good diagnostic performance. However, in order to use this model for diagnostic purposes, it should be tested at the appropriate medical center. This approach will allow the sample size to be significantly increased and improve the quality of the model. Since multi-chaotic analysis revealed the difference in multi-chaotic behavior within the pathological group, this allowed us to build a preliminary three-factor logistic model that is able to distinguish the chf from the af signal. This model has demonstrated a good performance, which makes this approach promising for building an expert system to discriminate between congestive heart failure and atrial fibrillation signals. However, to study the diagnostic characteristics of this approach, additional research with a larger sample is required.

In our study, multi-chaotic analysis exhibited a good performance, allowing the underlying dynamics of the system that generates the heart beat to be examined. This approach has a good potential for being used in the construction of expert systems for the diagnosis of cardiac pathologies.

## Figures and Tables

**Table 1:** The chaotic dynamical systems that were used to evaluate the generalized largest Lyapunov exponents (*GLLE*).

System	Equations	Parameters	Δt(s)	Theoretical LLE [20]
Logistic	$x_{i+1}=\mu x_{i}(1-x_{i})$	μ = 4.0	1	0.69
Henon	$x_{i+1}=1-ax_{i}^{2}+y_{i}$ $y_{i+1}=bx_{i}$	*a* = 1.4 *b* = 0.3	1	0.42
Lorenz	$x^{\prime }=\sigma (y-x)$ $y^{\prime }=x(R-z)-y$ $z^{\prime }=xy-bz$	σ = 10.0 *R* = 28 *b* = 8/3	0.01	1.50
Rossler	$x^{\prime }=-y-z$ $y^{\prime }=x+ay$ $z^{\prime }=b+z(x-c)$	*a* = 0.15 *b* = 0.20 *c* = 10.0	0.1	0.09

**Table 2:** Calculation results of *GLLE(p)* for chaotic dynamical systems.

*p*-Norm	Logistic, *GLLE(p)*	Henon, *GLLE(p)*	Lorenz, *GLLE(p)*	Rossler, *GLLE(p)*
0.1	0.69	0.42	1.44	0.07
0.5	0.69	0.43	1.50	0.09
1	0.69	0.43	1.51	0.09
2	0.69	0.42	1.52	0.09
3	0.67	0.42	1.51	0.09
4	0.62	0.39	1.53	0.09
5	0.49	0.34	1.52	0.09
6	0.38	0.30	1.30	0.06
7	0.29	0.25	1.25	0.06
8	0.25	0.22	1.32	0.06
9	0.22	0.18	1.26	0.06
10	0.22	0.16	1.27	0.06
Δ *W*	0.47	0.27	0.28	0.02

**Table 3:** Calculation results of the estimation of the spectrum width Δ*W* for chaotic dynamical systems with the addition of a random component, having a normal distribution with a zero mean and different standard deviations, to the x-coordinate time series.

Standard Deviation	Logistic, Δ*W*	Henon, Δ*W*	Lorenz, Δ*W*	Rossler, Δ*W*
0.001	0.50	0.31	0.26	0.01
0.01	0.28	0.23	0.21	0.01
0.05	0.20	0.17	0.21	0.01

**Table 4:** Median of the largest Lyapunov exponent *GLLE(2)* and the estimation of the spectrum width Δ*W* (*Me*—median, $Q_{1}$—first quartile, and $Q_{3}$—third quartile).

Group	Number	GLLE(2), $\mathrm{Me}(Q_{1}$$–Q_{3})$	Δ *W* $\mathrm{Me}(Q_{1}$ $–Q_{3})$
hs	54	0.11(0.10–0.13) ${}^{a}$	1.28(0.78–1.95) ${}^{c}$
chf	44	0.15(0.12–0.20) ${}^{a}$^,^${}^{b}$	2.61(1.70–4.23) ${}^{c}$^,^${}^{d}$
af	25	0.08(0.07–0.10) ${}^{a}$^,^${}^{b}$	0.42(0.26–0.79) ${}^{c}$^,^${}^{d}$

^*a*^ Significant difference between the *GLLE(2)* of healthy (hs) and pathological groups, *p*-value < 0.01 using the Kruskal–Wallis test; ^*b*^ significant difference between the *GLLE(2)* of congestive heart failure (chf) and atrial fibrillation (af) groups, *p*-value < 0.01, where a Kruskal–Wallis test was conducted; ^*c*^ significant difference between the Δ*W* of healthy (hs) and pathological groups, *p*-value < 0.01, using the Kruskal–Wallis test; ^*d*^ significant difference between the Δ*W* of congestive heart failure (chf) and atrial fibrillation (af) groups, *p*-value < 0.01, using the Kruskal–Wallis test.

**Table 5:** Median of the correlation dimension $D_{2}$ (*Me*—median, $Q_{1}$—first quartile, and $Q_{3}$—third quartile).

Group	Number	$D_{2},$ $\mathrm{Me}\;(Q_{1}$ $–Q_{3})$
hs	54	0.57(0.50–0.66) ${}^{a}$
chf	44	0.55(0.17–0.85) ${}^{b}$
af	25	0.93(0.69–1.00) ${}^{a}$^,^${}^{b}$

^*a*^ Significant difference betweenthe $D_{2}$ of healthy (hs) and atrial fibrillation (af) groups, *p*-value < 0.01, using the Kruskal–Wallis test, and ^*b*^ significant difference betweenthe $D_{2}$ of congestive heart failure (chf) and atrial fibrillation (af) groups, *p*-value < 0.01, using the Kruskal–Wallis test.

**Table 6:** The coefficients of the logistic regression five-factor model-1.

Value	Regression Coefficients b ± m	*p*-Value	Odds Ratio (95% CI)
GLLE_0.1	1.02 ± 0.28	<0.01	2.78 (1.59–4.84)
GLLE_0.5	−5.18 ± 2.11	<0.01	5.62 × 10${}^{-3}$(8.88 × 10${}^{-5}$ − 3.55 × 10${}^{-1}$)
GLLE_1	−7.09 ± 3.53	<0.01	8.29 × 10${}^{-4}$(8.11 × 10${}^{-7}$ − 8.47 × 10${}^{-1}$)
GLLE_4	105.39 ± 29.21	<0.01	5.92 × 10${}^{45}$(7.96 × 10${}^{20}$ − 4.41 × 10${}^{70}$)
GLLE_5	−95.23 ± 26.57	<0.01	4.37 × 10${}^{-42}$(1.0 × 10${}^{-64}$ − 1.84 × 10${}^{-19}$)
Constant	−0.45 ± 1.89	<0.01	

**Table 7:** Classification results of model-1.

Classification Results	Set			
	Training		Testing	
	Classification			
	Healthy Group	Pathological Group	Healthy Group	Pathological Group
Correct	31	39	11	20
Incorrect	8	7	4	3
Total cases	39	36	15	23

**Table 8:** The coefficients of the logistic regression three-factor model-2.

Value	Regression Coefficients b ± m	*p*-Value	Odds Ratio (95% CI)
GLLE_1	−4.851 ± 1.95	<0.01	7.82 × 10${}^{-3}$(1.73 × 10${}^{-4}$ − 3.55 × 10${}^{-1}$)
GLLE_4	75.71 ± 31.29	<0.01	7.62 × 10${}^{32}$(1.77 × 10${}^{6}$ − 3.28 × 10${}^{59}$)
GLLE_5	−70.84 ± 29.37	<0.01	1.71 × 10${}^{-31}$(1.72 × 10${}^{-56}$ − 1.69 × 10${}^{-6}$)
Constant	3.34 ± 3.01	<0.01	

**Table 9:** Classification results of model-2.

Classification Results	Set	
	Congestive Heart Failure Group	Atrial Fibrillation Group
Correct	40	19
Incorrect	4	6
Total cases	44	25

**Figure A1:**
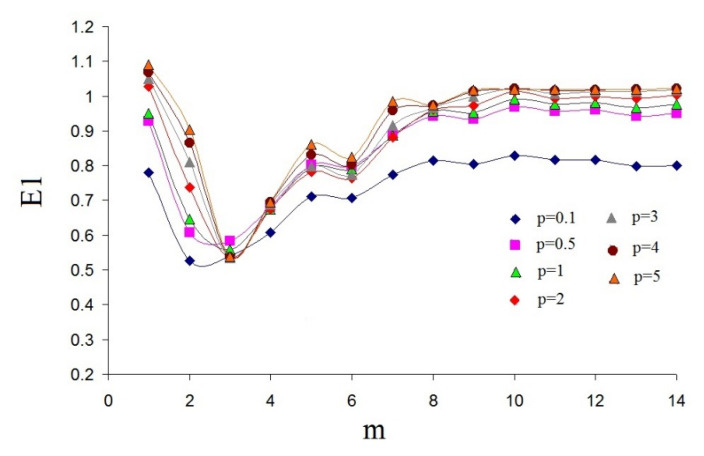
The dependencies of *E1(m)* versus m for R-R interval time series data for *p*-norm = 0.1, 0.5, and 1–5.

## Data Availability

Not applicable.

## Appendix A

For estimating embedding dimension *m*, we applied the method in [24].This method is based on the idea that increasing the dimension of the phase space reconstruction leads to a decreasing number of trajectory self-crossings and false neighbor decreases. In this method, a certain estimated *E1* value is calculated, which reaches a plateau if the required embedding dimension is achieved (for more details, see [24]). Figure A1 shows the plots of *E1 (m)* for different *p*-norm ${\left\|\right\|}_{p}$ for the R-R interval time series (Normal Sinus Rhythm RR Interval Database—nsr002).

As can be seen from the graph, the optimal embedding dimension is *m* = 9. This did not reveal a change in the optimal embedding dimension *m* with a changing *p*-norm ${\left\|\right\|}_{p}$.
